# The Localization of Cell Wall Components in the Quadrifids of Whole-Mount Immunolabeled *Utricularia dichotoma* Traps

**DOI:** 10.3390/ijms25010056

**Published:** 2023-12-19

**Authors:** Bartosz J. Płachno, Małgorzata Kapusta

**Affiliations:** 1 Department of Plant Cytology and Embryology, Institute of Botany, Faculty of Biology, Jagiellonian University in Kraków, 9 Gronostajowa St., 30-387 Cracow, Poland; 2 Laboratory of Bioimaging, Faculty of Biology, University of Gdańsk, 59 Wita Stwosza St., 80-308 Gdańsk, Poland; malgorzata.kapusta@ug.edu.pl

**Keywords:** arabinogalactan proteins, bladderworts, carbohydrate epitopes, carnivorous plants, cell wall, digestive glands, Lentibulariaceae, trichomes

## Abstract

*Utricularia* (bladderworts) are carnivorous plants. They produce small hollow vesicles, which function as suction traps that work underwater and capture fine organisms. Inside the traps, there are numerous glandular trichomes (quadrifids), which take part in the secretion of digestive enzymes, the resorption of released nutrients, and likely the pumping out of water. Due to the extreme specialization of quadrifids, they are an interesting model for studying the cell walls. This aim of the study was to fill in the gap in the literature concerning the immunocytochemistry of quadrifids in the major cell wall polysaccharides and glycoproteins. To do this, the localization of the cell wall components in the quadrifids was performed using whole-mount immunolabeled *Utricularia* traps. It was observed that only parts (arms) of the terminal cells had enough discontinuous cuticle to be permeable to antibodies. There were different patterns of the cell wall components in the arms of the terminal cells of the quadrifids. The cell walls of the arms were especially rich in low-methyl-esterified homogalacturonan. Moreover, various arabinogalactan proteins also occurred. Cell walls in glandular cells of quadrifids were rich in low-methyl-esterified homogalacturonan; in contrast, in the aquatic carnivorous plant *Aldrovanda vesiculosa*, cell walls in the glandular cells of digestive glands were poor in low-methyl-esterified homogalacturonan. Arabinogalactan proteins were found in the cell walls of trap gland cells in all studied carnivorous plants: *Utricularia*, and members of Droseraceae and Drosophyllaceae.

## 1. Introduction

The genus *Utricularia* is the most diverse genus among carnivorous plants, both in terms of plant size and environmental adaptations, as well as plant body architecture. It is also the richest in number of species, with around 250 species [1,2,3,4]. All of the *Utricularia* species are rootless herbs. Their vegetative organs go beyond the typical organ classification, i.e., either ‘root’, ‘stem’, or ‘leaf’, and they have intermixed morphological traits and developmental programs [5,6,7,8]. These are related to the heterotopic transfer of the function of the genes to other organs, as was shown in the root genes [9]. *Utricularia* produce small hollow vesicles (bladders with elastic walls and a mobile trap door), which function as suction traps that work underwater and capture fine organisms [10,11,12,13,14,15,16]. There is agreement that these traps are of a foliar origin. Using *Utricularia gibba*, Whitewoods et al. [17] showed that simple shifts in gene expression may change leaf morphogenesis, which finally results in trap formation. *Utricularia* traps attract special interest because they are among the fastest moving plant organs [15,18,19,20,21,22,23]. Poppinga et al. [23] showed that animals were successfully captured by *Utricularia australis* traps within 9 ms.

There are various types of glandular trichomes on the surface of the *Utricularia* traps [24,25,26,27,28,29]. The inner part of the trap is densely lined by two types of large trichomes. There are trichomes with four terminal cells called quadrifids, which almost cover the entire inner surface, and trichomes with two terminal cells called bifids, which are located near the trap door. Darwin proposed the names of these trichomes [30]. Both quadrifids and bifids have the same architecture, because are formed by a basal cell, a pedestal cell, and a terminal cell. The terminal cell is regionally differentiated and consists of different parts with distinct structures and functions: the basal part, stalk and arm. Quadrifids participate in the secretion of digestive enzymes and in the resorption of released nutrients, and probably also participate in pumping out water. According to some authors, the main role of bifids is pumping out water [26,27,31,32,33]. Due to the extreme specialization of the quadrifids, they are an interesting model to use to study cell walls. Recently, in a series of works, we showed the major cell wall polysaccharides and glycoproteins in the various glands of carnivorous plants; however, our studies were limited to *Aldrovanda vesiculosa* [34,35], *Dionaea muscipula* [36,37], and *Drosophyllum lusitanicum* [38]. This aim of this study is to fill in the gap in the literature concerning the immunocytochemistry of the quadrifids in the major cell wall polysaccharides and glycoproteins. Plant organs are covered by a cuticle, which is a hydrophobic and protective barrier, but is also impermeable to antibodies. However, structures such as the root hairs and pollen tubes are devoid of a cuticle, which means that the cell walls can be studied using whole-mount immunolabeled organs (without the time-consuming task of embedding them in resin and later cutting the material with a microtome). The use of this technique has produced very good results in root hairs [39,40,41] and pollen tubes [42,43]. Because cuticle discontinuities in the glands of carnivorous plants are known [26,27,44], we wanted to try this method; therefore, we used whole-mount immunolabeled *Utricularia* traps. However, to what extent a cuticle with discontinuities would be a barrier to the antibodies in these traps was an open question.

## 2. Results

### 2.1. Trap Morphology and Structure of a Quadrifid (Figure 1A–F)

**Figure 1:**
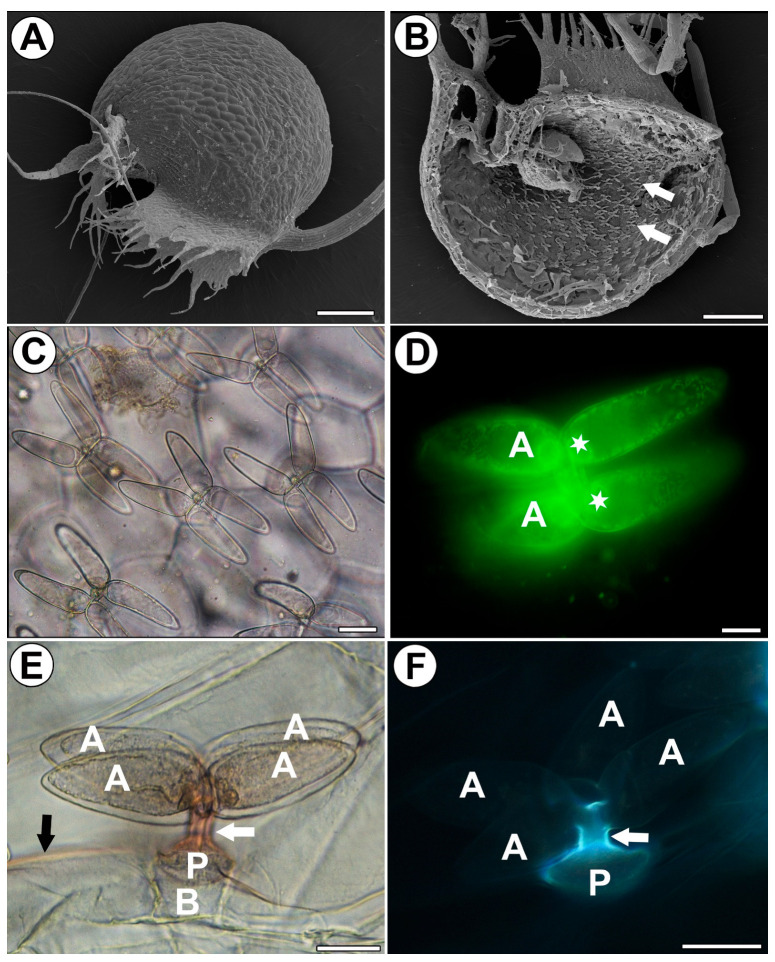
Distribution and morphology of the quadrifids of *Utricularia dichotoma* subsp. *novae-zelandiae*. (**A**) Morphology of a trap via SEM, scale bar 500 µm. (**B**) A sagittally halved trap showing the quadrifids (arrow), SEM, scale bar 500 µm. (**C**) Morphology of the quadrifids via light microscopy, scale bar 20 µm. (**D**) Staining with DiOC_6_ showed numerous mitochondria and nuclei (star) in terminal cells of a quadrifid, arm (A), via fluorescent microscopy, scale bar 10 µm. (**E**) Lateral view of quadrifid. Staining with Sudan (orange color); note the positive staining of cutin in stalks (white arrow) and cuticle of epidermis (black arrow); basal cell (B), pedestal cell (P), and arm (A) via light microscopy, scale bar 20 µm. (**F**) Lateral view of quadrifid; note the strong autofluorescence of cutin (blue–white) in stalks (white arrow) and the pedestal cell (P), but no autofluorescence of cutin in arms (A) via fluorescent microscopy, scale bar 20 µm.

A quadrifid has a typical structure and consists of a basal cell, pedestal cell (endodermoid) and four glandular cells. The glandular cells consist of a basal part, a stalk, and an arm (Figure 1C–F). The arm (the middle and distal regions) is highly vacuolated (Figure 1C,D). A large vacuole is surrounded by peripheral cytoplasm with numerous mitochondria. The cytoplasm of the arm is mostly concentrated towards its base, where the nucleus is also located (Figure 1D). Staining with Sudan III shows positive result of cutin occurrence in stalks of trichome terminal cells, the pedestal cell, and trap epidermis (Figure 1E). An autofluorescence of cutin occurs in stalks of trichome terminal cells and the pedestal cell (Figure 1F). Similar results were obtained using staining with Auramine O (Figure S1A).

### 2.2. Distribution of the Arabinogalactan Proteins (AGPs) 

**Figure 2:**
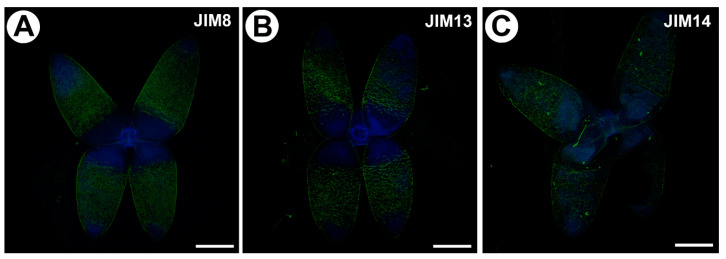
Arabinogalactan proteins detected in the quadrifids of *Utricularia dichotoma* subsp. *novae*-*zelandiae*. Green fluorescence is a signal of antibodies. Blue fluorescence shows the autofluorescence of the cell wall and cuticle. (**A**) Arabinogalactan proteins (labeled with JIM8) were detected in the quadrifid, scale bar 10 µm. (**B**) Arabinogalactan proteins (labeled with JIM13) were detected in the quadrifid, scale bar 10 µm. (**C**) Arabinogalactan proteins (labeled with JIM14) were detected in the quadrifid, scale bar 10 µm.

We used the JIM8, JIM13 and JIM14 antibodies in order to localize the AGPs (Figure 2A–C). JIM8 reacted with fluorescence in the cell walls of arms of the quadrifids (Figure 2A), but only in the apical and middle parts of the arms. JIM13 gave a fluorescence signal of the AGP epitopes in the cell walls of the arms of the quadrifids (Figure 2B), but not in the basal part. The signal was intense in the middle parts of the arms, and less intense in the apical part of the arms. JIM14 reacted with fluorescence in the cell walls of the arms of the quadrifids (Figure 2C), but only in the apical and middle parts of the arms.

### 2.3. Distribution of Homogalacturonan 

**Figure 3:**
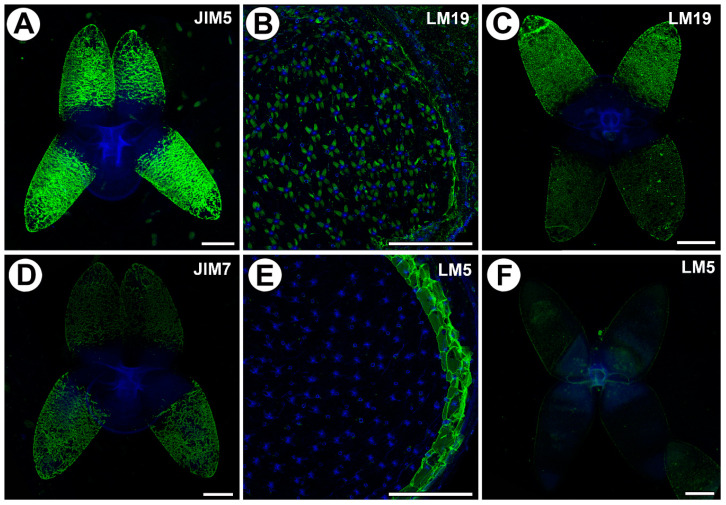
Homogalacturonan (HG) detected in the in the quadrifids of *Utricularia dichotoma* subsp. *novae-zelandiae*. Green fluorescence is a sign of antibodies. Blue fluorescence indicates autofluorescence of the cell wall and cuticle. (**A**) HG (labeled with JIM5) detected in the quadrifids, scale bar 10 µm. (**B**) HG (labeled with LM19) detected in the quadrifids, scale bar 500 µm. (**C**) HG (labeled with LM19) detected in the quadrifids, scale bar 10 µm. (**D**) HG (labeled with JIM7) detected in the quadrifids, scale bar 10 µm. (**E**) HG (labeled with LM5) detected in the trap; note the blue autofluorescence of quadrifids inside the trap and the strong green fluorescence signal of antibodies in the cell walls of the trap wall, scale bar 500 µm. (**F**) HG (labeled with LM5) detected in the quadrifid, scale bar 10 µm.

Low-methyl-esterified homogalacturonans (HGs) were detected by the JIM5 and LM19 antibodies (Figure 3A–C). Both JIM5 and LM 19 reacted with a strong fluorescence in the cell walls of the arms of the quadrifids (Figure 3A,C), but only in the apical and middle parts of the arms.

Highly esterified HGs were detected by the JIM7 antibodies. Fluorescence signals were observed in the cell walls in the apical and middle parts of the arms of the quadrifids (Figure 3D). The pectic polysaccharide (1–4)-β-d-galactan was detected by LM5 (Figure 3E,F). The arms of the quadrifids gave only a weak signal (Figure 3F). However, LM5 reacted with a strong fluorescence in the cell walls of the sectioned cells of the trap wall (Figure 3E).

### 2.4. Distribution of Hemicellulose 

**Figure 4:**
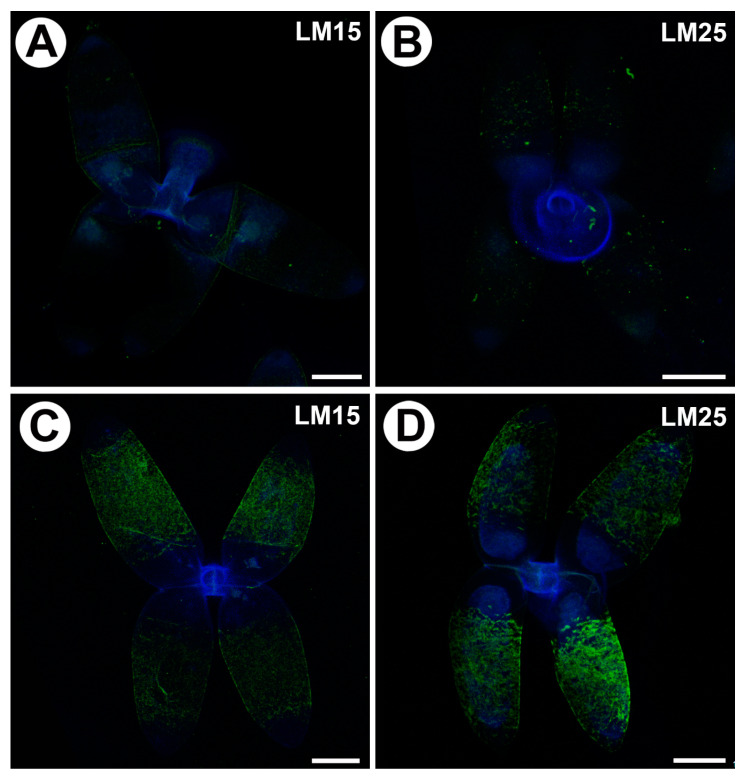
Xyloglucan detected in the quadrifids of *Utricularia dichotoma* subsp. *novae*-*zelandiae*. Green fluorescence is a sign of antibodies. Blue fluorescence indicates autofluorescence of the cell wall, cuticle and vacuole. (**A**) Xyloglucan (labeled with LM15) detected in the quadrifids, scale bar 10 µm. (**B**) Xyloglucan (labeled with LM25) detected in the quadrifids, scale bar 10 µm. (**C**) Xyloglucan (labeled with LM15) detected in the quadrifids after they had been pre-treated with pectate lyase, scale bar 10 µm. (**D**) Xyloglucan (labeled with LM25) detected in the quadrifids after they had been pre-treated with pectate lyase, scale bar 10 µm.

Xyloglucan was detected by the LM15 and LM25 antibodies. Both gave a very weak fluorescence signal in the cell walls of the arms of the quadrifids (Figure 4A,B). When the sections were pre-treated with pectate lyase (during which pectins were removed), the LM15 antibody gave a stronger fluorescence signal (Figure 4C); similarly, the antibody signal was stronger in the case of LM25 (Figure 4D).

## 3. Discussion

### 3.1. Pros and Cons of the Whole-Mount Immunolabeled Trap Technique

Immunolabeling was not successful in the outer and inner epidermis of the traps. Immunolabeling was successful in the cell walls of the sectioned cells of the trap wall (outer and inner epidermis of the trap), where the cuticle was mechanically damaged and the cell wall was exposed. In the quadrifids, this method gave good results only in the terminal cells. Here, the antibodies penetrated the cell wall only in the middle and apical parts of the arms. Our results can be better understood if the formation and structure of the cuticle in *Utricularia* traps is considered. Fineran and Lee [25] described the ultrastructure of *Utricularia* trap cells in detail. They showed that the *Utricularia* trap epidermal cells have a developed cuticle. Moreover, the pedestal cell of the quadrifids has a cuticle and the lateral wall of the pedestal cell is also impregnated with cutin. According to these authors, the outermost region in the wall of the stalk (part of the terminal cell) is also heavily impregnated with an opaque cuticular material. Cuticular impregnation also occurs at the base of the arms. This would explain why immunolabeling was unsuccessful in these regions. However, in the middle and apical parts of the arm, the cuticle had a more open structure [25]; thus, the cuticle was permeable for antibodies, as we showed. The whole-mount immunolabeled organ technique enabled the dehydration, resin infiltration, and material slicing using a microtome to be avoided, which saved time and reagents. However, in our case, this technique should be regarded as a good preliminary method for quickly assessing the cell wall components in the most exposed cells with a discontinuous cuticle. Carnivorous plants have differently developed cuticles in their glandular structures. These cuticles are permeable because they have discontinuities such as cuticular gaps, cuticular pores, a ruptured cuticle, the open structure of cuticle, and a loose cuticle that is composed of cuticular droplets, e.g., [44,45,46,47,48,49,50,51]. Future research should focus on how these differentially formed cuticular discontinuities permit antibody penetration.

### 3.2. Cell Wall Polymers and Quadrifid Activity 

The cell wall polymers are dynamic components and can be restructured and redistributed during plant development, reproduction, and organ aging [52,53,54,55,56,57]. These components play important roles during stresses and plant interactions with other organisms, e.g., bacteria and fungi [58,59,60,61,62]. *Utricularia* quadrifids are not only quite specialized trichomes in their structure; they also perform various functions, e.g., secretion of digestive enzymes, resorption of released nutrients, and participation in pumping out water [25,26,44]. Enzymes such as protease, phosphatase, and esterase were localized cytochemically in the quadrifids [31,32,63]. Aminopeptidase, phosphatase, β-hexosaminidase, α- and β-glucosidase were found in *Utricularia* trap fluid [64,65]. These trichomes are also active in respiration. Adamec [66] demonstrated the great respiratory activity of *Utricularia* traps and their trichomes immediately after firing, which results in the formation of anoxia, which kills prey inside the traps. Inside aquatic *Utricularia* traps, a rich community of commensal microorganisms exists, including bacteria, cyanobacteria, microfungi, algae, protozoa, and rotifers [67,68,69]. Thus, the quadrifids are also exposed to stress that is not only associated with contact with captured organisms, but also with a number of organisms that are living as commensals or parasites in the traps. For the above reasons, the presence of AGPs in the cell wall of the secretory cells of the quadrifids is not surprising, especially since these cells are responsible for the secretion of enzymes and subsequent nutrient intake, and these processes involve vesicle trafficking and membrane recycling, in which AGPs participate [70,71,72]. Moreover, the cell walls of the secretory cells in the glands of other carnivorous plants such as *Drosophyllum* [38], *Dionaea* [34,35] and *Aldrovanda* [36,37] are enriched with arabinogalactan proteins. We found that the cell walls of the arms are especially rich in low-methyl-esterified homogalacturonans. It is known that homogalacturonans are involved in plant cell wall porosity, elasticity, and hydration [73,74]. These features are important for transporting substances through the cell wall. Therefore, the presence of these pectins in the cell wall of glandular cells in quadrifids may be crucial for gland function (secretion of digestive enzymes, and later, absorption of nutrients from digested invertebrate bodies). However, to prove this, it would be necessary to study a mutant of *Utricularia* with a disruption of pectin production. However, in the glands of carnivorous plants from the families Droseraceae and Drosophyllaceae that have been studied to date, the cell walls of secretory cells have been poor in low-methyl-esterified homogalacturonans (as detected by the JIM5 and LM19 antibodies) [34,35,38]. One can only speculate about whether these differences are related to the phylogenetic position of the species or to the functioning of the glands. In the glands of carnivorous plants from the families Droseraceae and Drosophyllaceae that have been studied to date, the cell walls of the secretory cells are enriched with hemicelluloses (detected by the LM15 and LM25 antibodies, which both recognize xyloglucan). We found that in quadrifids, pectins mask the presence of hemicelluloses [75]. Hemicelluloses contribute to strengthening the cell wall by interaction with cellulose [76,77]; their increased presence may be related to the exposure of trichome cells to damage by organisms. We think that to better understand both the cell wall polymers and gland activity in *Utricularia*, samples cryo-fixed by high-pressure freezing and immunogold techniques are required, particularly since the method we used allows us to examine the components of the wall only to a limited extent. We do not know to what depth of the cell wall the penetration by antibodies reaches.

Future research should examine the composition of *Utricularia* cell walls in various vegetative organs, and compare this composition not only with trichome cell walls, but also with trap epidermal cells. Since there has been clear progress in genome analysis in *Utricularia* [9,78,79,80,81], we have hope for a future better understanding of how cell wall composition plays a role in trap function.

## 4. Materials and Methods

### 4.1. Plant Material

*Utricularia dichotoma* subsp. *novae-zelandiae* (Hook.f) R.W.Jobson [82] plants were grown in the greenhouses of the Botanical Garden of Jagiellonian University. The plants were cultivated in wet peat and exposed to natural sunlight.

### 4.2. Histological and Immunochemical Analysis

The traps were hand-sliced into two halves using a razor blade, and fixed in 8% (*w*/*v*) paraformaldehyde (PFA, Sigma-Aldrich, Sigma-Aldrich Sp. z o.o., Poznań, Poland) mixed with 0.25% (*v*/*v*) glutaraldehyde (GA, Sigma-Aldrich, Sigma-Aldrich Sp. z o.o., Poznań, Poland) in a PIPES buffer overnight at 4 °C. The PIPES buffer contained 50 mM PIPES (piperazine-*N*,*N*′-bis [2-ethanesulfonic acid], Sigma-Aldrich, Sigma-Aldrich Sp. z o.o., Poznań, Poland), 10 mM EGTA (ethylene gly col-bis[β-aminoethyl ether]*N*,*N*,*N*′,*N*′-tetraacetic acid, Sigma Aldrich, Poznań, Poland), and 1 mM MgCl_2_ (Sigma-Aldrich, Sigma-Aldrich Sp. z o.o., Poznań, Poland), pH 6.8. The plant material was washed in a PBS buffer and later blocked with 1% bovine serum albumin (BSA, Sigma-Aldrich) in a PBS buffer and incubated with the following primary antibodies (Table 1) [83]: anti-AGP: JIM8, JIM13, and JIM14 [84,85,86,87]; anti-pectin: JIM5, JIM7, LM19, and LM5 [75,84,88,89]; and anti-hemicelluloses: LM25 and LM15 [75,88,89], overnight at 4 °C. All of the primary antibodies were used in a 1:20 dilution. They were purchased from Plant Probes, Leeds, UK, and the goat anti-rat secondary antibody conjugated with FITC was purchased from Abcam (Abcam plc, Cambridge, UK). The signal of antibodies was visualized using laser 488 emission 498–550 (green fluorescence), and the autofluorescence of cell walls and cutin was visualized with laser 405 emission 415–460 (blue fluorescence). The samples were then cover-slipped using a Mowiol mounting medium (a mixture of Mowiol^®^4-88 (Sigma-Aldrich, Sigma-Aldrich Sp. z o.o., Poznań, Poland) and glycerol for fluorescence microscopy (Merck, Warsaw, Poland)), with the addition of 2.5% DABCO (The Carl Roth GmbH + Co. KG, Karlsruhe, Germany). The plant material was viewed using a Leica STELLARIS 5 WLL confocal microscope (Wetzlar, Germany) with lightning deconvolution. At least two different replications were performed for each of the analyzed traps, and about five to ten trichomes from each trap were analyzed for each antibody that was used. Negative controls were created by omitting the primary antibody step, which caused no fluorescence signal in any of the control frames for any of the stained slides (Figure S1B). To remove the HG from the cell walls, the traps were pretreated with 0.1 M sodium carbonate pH = 11.4 for 2 h at room temperature. This was followed by digestion with a pectate lyase 10 A (Nzytech) at 10 μg/mL for 2 h at room temperature in 50 mM N-cyclohexyl-3-aminopropane sulfonic acid (CAPS) with the addition of 2 mM of a CaCl_2_ buffer at pH 10 [38], and then incubation with the JIM5, JIM7, LM15, and LM25 antibodies, as described above.

### 4.3. Light Microscopy (LM) 

The fluorescence of the mitochondria and nuclei in the quadrifids was observed by staining the traps with 20 μM DiOC_6_ (3,3′-dihexyloxacarbocyanine iodide; Thermo Fisher, Rockland, MD, USA) dissolved in water [90]. The traps were examined using a Nikon Eclipse E400 light microscope. Cutin and cuticle were stained using Sudan III (Sigma-Aldrich Sp. z o.o., Poznań, Poland) and Auramine O (Sigma-Aldrich Sp. z o.o., Poznań, Poland), and later, traps were examined using a Nikon Eclipse E400 light microscope (Tokyo, Japan) with a UV-2A filter (Ex. 330–380 nm, DM. 400 nm, Em. 420-α nm).

### 4.4. Morphological Observation

For the scanning electron microscopy (SEM), the traps were fixed in methanol and later transferred to ethanol, and then transferred to acetone and dried using supercritical CO_2_. The material was then sputter-coated with gold and examined at an accelerating voltage of 20 kV using a Hitachi S-4700 scanning electron microscope (Tokyo, Japan), which is housed at the Institute of Geological Sciences, Jagiellonian University, Kraków, Poland [38].

## 5. Conclusions

The advantage of analyzing whole traps without dehydrating, resin infiltrating, and slicing them using a microtome is that it saves time and reagents. However, our analyses have shown that in intact cells, only those parts of the cells that have a discontinuous cuticle gave positive results.

Here, we show that the cell walls of the secretory cells of the quadrifids were enriched with low-methyl-esterified homogalacturonans is in contrast to the cell walls of gland cells of carnivorous species from the Droseraceae and Drosophyllaceae families. However, a common characteristic of the secretion of these glands from the traps of carnivorous plants is the occurrence of arabinogalactan proteins in the cell walls, which may indirectly indicate the important role of these components in the functioning of glandular structures.

## Figures and Tables

**Table 1:** List of the monoclonal antibodies used in the current study, and the epitopes they recognize [83].

Antibody	Epitope
AGPs	
JIM8	Arabinogalactan
JIM13	Arabinogalactan/arabinogalactan protein
JIM14	Arabinogalactan/arabinogalactan protein
Homogalacturonan	
JIM5	Homogalacturonan (HG) domain of c pectic polysaccharides; recognizes partially methyl-esterified epitopes of HG, and can also bind to unesterified HG
JIM7	HG domain of the pectic polysaccharides; recognizes partially methyl-esterified epitopes of HG, but does not bind to unesterified HG
LM5	Linear tetrasaccharide in (1–4)-β-d-galactans (RGI side chain)
LM19	HG domain in pectic polysaccharides; recognizes a range of HG with a preference to bind strongly to unesterified HG
	Hemicelluloses
LM15	XXXG motif of xyloglucan
LM25	XLLG, XXLG and XXXG motifs of xyloglucan

## Data Availability

The data presented in this study are available on request from the corresponding author.

## Supplementary Materials

The following supporting information can be downloaded at: https://www.mdpi.com/article/10.3390/ijms25010056/s1.

## Abbreviations

AGPs—arabinogalactan proteins, HG—homogalacturonans, SEM—scanning electron microscope, LM—light microscope.
