# Supplementary material for: The Localization of Cell Wall Components in the Quadrifids of Whole-Mount Immunolabeled Utricularia dichotoma Traps
Source: Int J Mol Sci. 2023 Dec 19;25(1):56. doi: 10.3390/ijms25010056 (PMC10778831; doi:10.3390/ijms25010056)

Supplementary Materials

Figure S1. (A) Lateral view of quadrifid. Staining with Auramine O (yellow fluorescence), note positive staining of cutin in stalks (white arrow) and cuticle of epidermis (red arrow); pedestal cell (P), arm (A), fluorescent microscopy, bar 20 μm. (B) Control reactions of cell wall components after immunolabeling in quadrifid; stalks (white arrow), pedestal cell (P), arm (A), confocal microscopy, bar 10 μm.

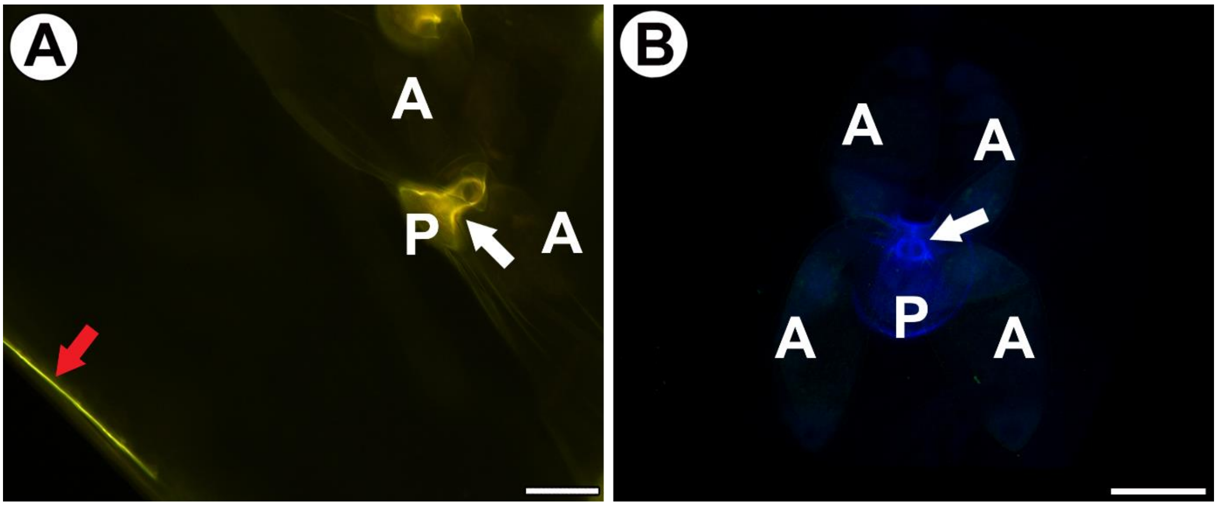

Supplement: Supplementary file 1 [file ijms-25-00056-s001.zip › ijms-2761377-supplementary.pdf]
